# Supplementary material for: The causal relationship between the human gut microbiota and pyogenic arthritis: a Mendelian randomization study
Source: Front Cell Infect Microbiol. 2024 Nov 26;14:1452480. doi: 10.3389/fcimb.2024.1452480 (PMC11629706; doi:10.3389/fcimb.2024.1452480)
Supplement: Supplementary file 1 [file DataSheet1.zip › mendelian test/ebi-a-GCST90027810.csv_forest.pdf]

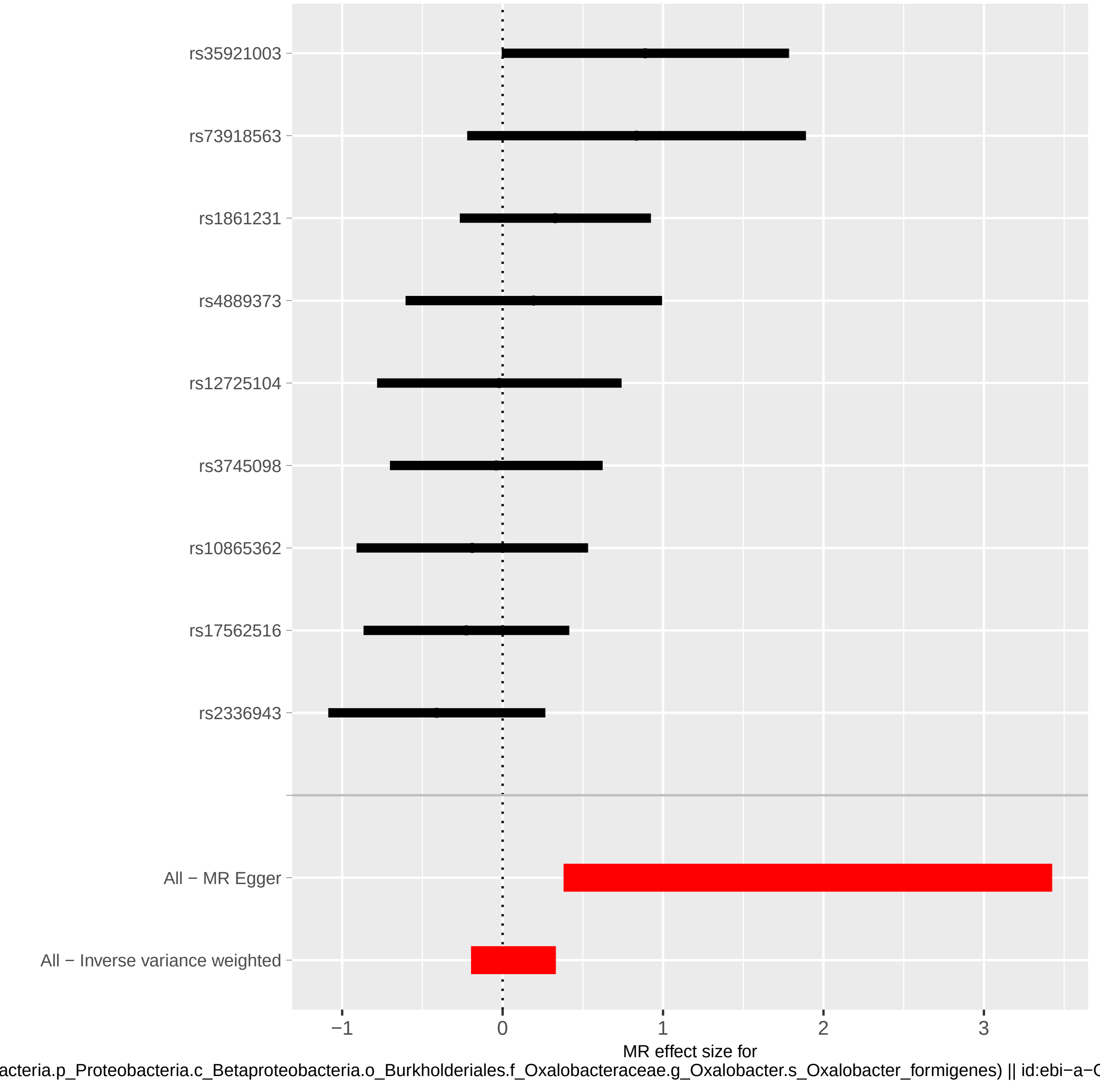

rs35921003
rs73918563
rs1861231
rs4889373
rs12725104
rs3745098
rs10865362
rs17562516
rs2336943
All − MR Egger
All − Inverse variance weighted
−1
0
1
2
3
MR effect size for
acteria.p_Proteobacteria.c_Betaproteobacteria.o_Burkholderiales.f_Oxalobacteraceae.g_Oxalobacter.s_Oxalobacter_formigenes) || id:ebi−a−G
